# Linking long non-coding RNAs and SWI/SNF complexes to chromatin remodeling in cancer

**DOI:** 10.1186/s12943-017-0612-0

**Published:** 2017-02-17

**Authors:** Yanyan Tang, Jinpeng Wang, Yu Lian, Chunmei Fan, Ping Zhang, Yingfen Wu, Xiayu Li, Fang Xiong, Xiaoling Li, Guiyuan Li, Wei Xiong, Zhaoyang Zeng

**Affiliations:** 1 The Key Laboratory of Carcinogenesis of the Chinese Ministry of Health, Xiangya Hospital, Central South University, Changsha, Hunan China; 2 The Key Laboratory of Carcinogenesis and Cancer Invasion of the Chinese Ministry of Education, Cancer Research Institute, Central South University, Changsha, Hunan China; 3 Hunan Key Laboratory of Nonresolving Inflammation and Cancer, Disease Genome Research Center, The Third Xiangya Hospital, Central South University, Changsha, Hunan China; 4 School of Information Science and Engineering, Central South University, Changsha, Hunan China

**Keywords:** Long non-coding RNAs (lncRNAs), The SWI/SNF complexes, Chromatin remodeling, Gene expression, Carcinogenesis

## Abstract

Chromatin remodeling controls gene expression and signaling pathway activation, and aberrant chromatin structure and gene dysregulation are primary characteristics of human cancer progression. Recent reports have shown that long non-coding RNAs (lncRNAs) are tightly associated with chromatin remodeling. In this review, we focused on important chromatin remodelers called the switching defective/sucrose nonfermenting (SWI/SNF) complexes, which use the energy of ATP hydrolysis to control gene transcription by altering chromatin structure. We summarize a link between lncRNAs and the SWI/SNF complexes and their role in chromatin remodeling and gene expression regulation in cancer, thereby providing systematic information and a better understanding of carcinogenesis.

## Background

Cancer is one of the leading causes of death in the world. Dysregulation of chromatin remodeling and cytoskeleton organization is tightly associated with the progression of cancer [1]. Chromatin remodeling is the dynamic modification of the chromatin architecture to allow regulatory transcription machinery proteins access to condensed genomic DNA and thereby control gene expression [2, 3]. Some specific protein complexes, which can move, eject, replace or restructure nucleosomes with energy from the hydrolysis of adenosine triphosphate (ATP), play a pivotal role in the process of chromatin remodeling by providing proper nucleosome position and density [4]. Two major classes of protein complexes control the process of chromatin remodeling. One class is the covalent histone-modifying complexes, and the other is the ATP-dependent chromatin remodeling complexes. Chromatin remodeling is currently a major therapeutic strategy in the treatment of several cancers.

In eukaryotes, according to the different ATP enzyme and protein subunits, the ATP-dependent chromatin remodeling complexes can be divided into four classes: switching defective/sucrose nonfermenting (SWI/SNF), imitation switch (ISWI), chromodomain helicase DNA binding (CHD), and inositol requiring 80 (INO80) [2]. There are large differences in the mechanisms of the four classes. The SWI/SNF complexes recognize the nucleosome and naked DNA with a high affinity and can move the nucleosome by interacting with DNA, resulting in a more easily affected DNA region [5]. SWI/SNF is usually associated with activated chromatin and can silence gene expression. ISWI recognizes only the nucleosome to silence chromatin with low activity. Some CHD complexes slide or eject nucleosomes to promote transcription, and others play repressive roles with histone deacetylases. INO80 can activate transcription and DNA repair [2].

Long non-coding RNAs (lncRNAs) are a subset of noncoding RNAs, which exceed 200 nucleotides in length [6–8]. Accumulating evidence has confirmed that lncRNAs contribute to cancer initiation and progression through regulating gene transcription and post-transcriptional regulation through chromatin remodeling [9–16]. In this review, we described the relationship between lncRNAs and the SWI/SNF complexes on the mechanism of transcriptional regulation and chromatin modification in cancer. This link will provide new insights for future studies on cancer.

## The structure and composition of the SWI/SNF complexes

The SWI/SNF complexes, which were first discovered in yeast, regulate the expression of homothallic switching endonuclease (HO) and the transforming enzyme sucrose invertase (SUC2) [17]. Among these complexes, HO is a mating type switch (SWI) and SUC2 is necessary for sucrose non-fermentation (SNF) [18, 19]. Therefore, the complexes have come to be known as the SWI/SNF complexes.

**Fig. 1:**
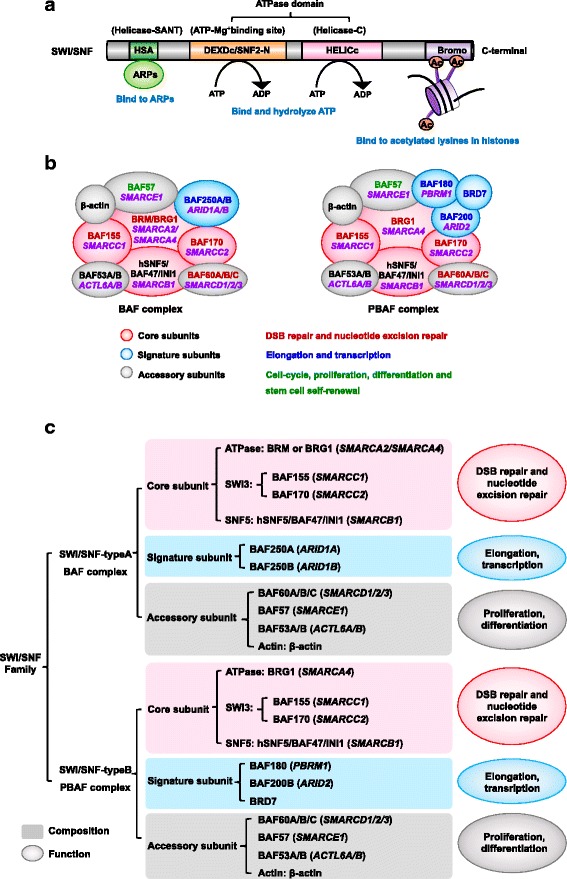
The structure and composition of the SWI/SNF complexes in human. **a** The structure of the SWI/SNF complexes. The SWI/SNF complexes contain a DNA-dependent ATPase as its catalytic subunit and distinct flanking domains, such as HSA and bromodomain. **b** The subfamily of the SWI/SNF complexes. The SWI/SNF complexes consist of several core subunits (*pink*), signature subunits (*blue*) and additional accessory subunits (*gray*). The subunit coded gene is marked in *purple with italics*. Different colors of characters in a complex indicate the function of various subunits. **c** The outline of the SWI/SNF complexes. The SWI/SNF complexes are divided into two types: *BAF* and *PBAF*. Two types of the SWI/SNF complexes contain different subunits, which are related to distinct functions

The SWI/SNF complexes contain a conserved DNA-dependent ATPase as their catalytic subunit and distinct flanking domains, such as helicase-SANT-associated domains (HSA) and bromodomain proteins [3]. The ATPase domain (core subunit) is composed of two parts: DEXDc and HELICc. DEXDc, also called SNF2-N, contains an ATP Mg^2+^ binding site and a motor unit called the DEAD box domain, which converts ATP energy to mechanical movement [20]. HSA can bind to ARPs and actin [2]. At the C-terminal, a bromodomain is located, which can recognize acetylated lysines in histones and contributes to increasing remodeling efficiency [2] (Fig. 1a).

Most remodeling enzymes form a multi-subunit complex to perform the remodeling function. The SWI/SNF complexes are divided into two sub-classes as distinguished by different subunits; one sub-class is the BRG/hBRM associated factors (BAF) complexes, and the other is the polybromo associated BAF (PBAF) complexes [3]. The two sub-classes of complexes are always composed of 8 to 14 subunits, including several core subunits (ATPase subunit), the BAF complex containing either Brahma (BRM) or BRM-related Gene 1 (BRG1), and the PBAF complex only containing BRG1 [21]. The other core components are BAF155 (*SMARCC1* gene encoded), BAF170 (*SMARCC2*) and SNF5/BAF47/INI1 (*SMARCB1*) in both the BAF and PBAF complexes. The core subunits are the most important subunits, possessing the same level of remodeling activity as the entire SWI/SNF complex [22, 23]. Core subunits are mostly involved in double-strand break (DSB) repair and nucleotide excision repair.

In addition to these core subunits, the signature subunits of the SWI/SNF complexes, which are associated with elongation and transcription, are BAF250A/B (*ARID1A/B*) in BAF complexes and BAF180 (*PBRM1*), BAF200 (*ARID2*) and BRD7 in PBAF complexes [4, 24]. Moreover, many accessory subunits are part of the SWI/SNF complexes, such as BAF57, BAF53A/B, BAF60A/B/C and β-actin. The function of each subunit is slightly different through conserved proteins, and unique attendant subunits distinguish each complex (Fig. 1b and c).

## The SWI/SNF complexes mediates chromatin remodeling

The hypothesized steps of chromatin remodeling include the recruitment and localization of the SWI/SNF complexes to target genes followed by the hydrolysis of ATP to release energy and alter chromatin structure. Early observations showed that there are three models of the SWI/SNF complexes recruited and located on chromatin [25]. The first is the “non-targeting model”, meaning that the SWI/SNF complexes locate anywhere on chromatin in a random manner, requiring only one DNA binding transcription factor. In this model, chromatin structure remodeling will occur, but this model seems inadequate to clarify the function of the SWI/SNF complexes [25]. The second model is “RNA polymerase II association”, in which the SWI/SNF complexes are associated with a specific nucleosome by RNA polymerase II [26]. The last model is “targeting by activators”, in which the SWI/SNF complexes are recruited to target genes by transcriptional activators and transcription factors [27].

There are two related models for the mechanism by which the SWI/SNF complexes remodel chromatin conformations. The “nucleosome slide model”, where the SWI/SNF complexes use the energy of ATP-hydrolysis to remodel histones, which results in changes in the chromatin structure. The SWI/SNF complexes can work as a DNA translocase enzyme. When the DNA double strand is opened, the SWI/SNF complexes can make the nucleosome slide along the DNA. In this way, the relative movement of histones and the DNA change the position of the nucleosome. At the same time, restricted enzyme sites of DNA are exposed, which results in transcription factors being bound to corresponding elements [28]. However, the slide model changes only the position of the nucleosome, which cannot explain how a large amount of exposed DNA can be formed in a closely packed region. Therefore, there may be some other mechanisms for chromatin remodeling. The other model is the “bulge model”, in which the SWI/SNF complexes push or pull linker DNA into nucleosome regions, resulting in the DNA bulge exposure or inhibition of a section of DNA sequence. No matter the assumptions, the DNA bulge will change the interaction of the histone octamer with the DNA, and the relative position of DNA on a histone will change and cause the sliding of the nucleosome on DNA. Eventually, chromatin remodeling mediated by the SWI/SNF complexes will cause activation or inhibition of the corresponding genes [28].

## LncRNAs interact with the SWI/SNF complexes regulating chromatin remodeling in cancer

The chromatinic structure can undergo dynamic changes in the cell. When the chromatic structure is compact, it will prevent transcription factors and RNA polymerase recruitment and binding to specific DNA sequences, leaving genes silenced. While the chromatinic structure is loose, transcription factors can bind to the promoter of a specific gene and activate transcription. The SWI/SNF complexes usually remodel chromatin in a loose state and are associated with the activation or suppression of gene expression [29]. SWI/SNF affects genes expression through chromatin status, such as nucleosome positioning, exchanging or moving [30, 31]. Recent studies showed that lncRNAs regulate chromatin modification and gene expression through their interactions with the SWI/SNF complexes. According to many reports, the interaction between lncRNAs and the SWI/SNF complexes can be divided into two models in cancer: the binding model and the recruiting model.

**Fig. 2:**
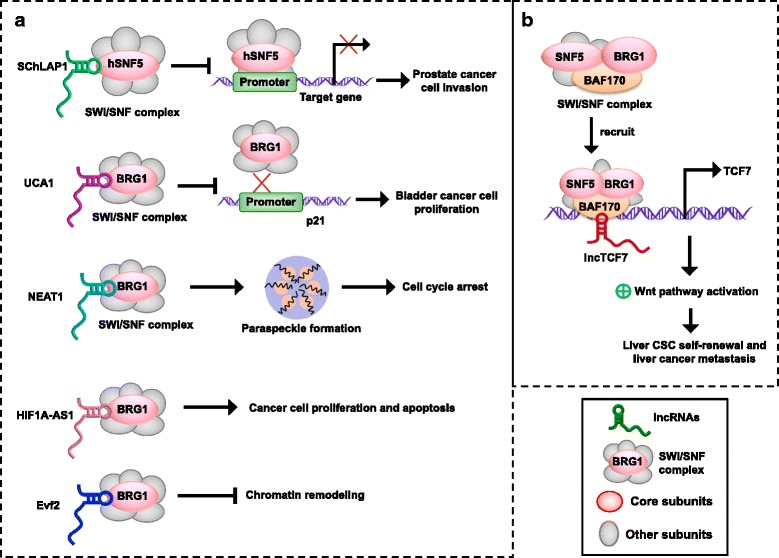
LncRNAs interact with the SWI/SNF complexes to regulate chromatin remodeling and cancer progression. **a**. Binding model, lncRNAs bind subunits of the SWI/SNF complexes to regulate target genes expression. **b**. Recruiting model, lncRNAs recruited SWI/SNF complexes or some core subunits to the target gene, thereby affecting the gene expression

The binding model states that lncRNAs can directly bind to the subunit of the SWI/SNF complexes and serve as a guide to anchor the SWI/SNF complexes or function as a decoy to keep chromatin modifiers away from specific genomic sites. Moreover, lncRNAs can be incorporated into the SWI/SNF complexes and function as a scaffold to assemble the complex for chromatin remodeling [32]. One such lncRNA is called Second Chromosome Locus Associated with Prostate 1 (*SChLAP1*), which is overexpressed in a subset of prostate cancers. The mechanism of *SChLAP1* is direct binding to hSNF5, which can antagonize tumor suppressive functions of the SWI/SNF complexes by decreasing their genomic binding. Therefore, *SChLAP1* promotes tumor cell invasion and metastasis by binding and impairing proper SWI/SNF regulation of gene expression [33, 34] (Fig. 2). The lncRNA *UCA1* can bind to BRG1, the core subunit of the SWI/SNF complexes, and damage the remodeling activity of BRG1, which reduces the expression of p21 by blocking BRG1 binding to the p21 promoter and promotes the proliferation of bladder cancer cells [35]. Nuclear paraspeckle assembly transcript 1 (*NEAT1*) is a nuclear-restricted lncRNA, which is dysregulated in various human cancers, including leukemia, bladder cancer, lung cancer, breast cancer and gastric cancer [36–40]. In the process of lncRNA-dependent nuclear body assembly, *NEAT1* directly interacts with the SWI/SNF core unit, BRG1 or BRM, to form the paraspeckle structure, which leads to cell cycle arrest and affects cancer progression [41, 42]. The lncRNA *Evf2* can directly bind to BRG1 through distinct binding sites and repress the activity of BRG1 ATPase and chromatin remodeling [43]. The lncRNA *HIF1A-AS1* binds to BRG1, which contributes to the regulation of cell proliferation and apoptosis in cancer [44, 45] (Fig. 2).

The recruiting model involves lncRNAs recruited SWI/SNF complexes or some core subunits to the target gene, thereby affecting the gene structure and expression. For instance, the lncRNA *lncTCF7* was overexpressed in hepatocellular carcinoma and promoted tumorigenesis of liver cancer stem cells. The mechanism is *lncTCF7* recruitment of the core subunits of the SWI/SNF complexes, BRG1, SNF5 and BAF170, to the TCF7 promoter. Furthermore, the SWI/SNF complexes trigger the TCF7 gene and promote tumor progression [46]. Another example of the recruiting model is RNA Polymerase V-generated lncRNA, which can guide the SWI/SNF complexes to genome specific loci [47]. These above lncRNAs show that lncRNAs regulate the carcinogenesis of cancer through interacting with different subunits of the SWI/SNF complexes in two different manners (Table 1 and Fig. 2).

**Table 1:** Summary of lncRNAs interacting with the SWI/SNF complex in cancer

LncRNAs	Interaction of lncRNA with SWI/SNF	Cancer type	Refs
*SChLAP1*	*SChLAP1* binds to hSNF5/BAF47 and antagonizes the tumor suppressive functions of the SWI/SNF complex.	Prostate cancer	[33, 34]
*LncTCF7*	*LncTCF7* recruits BRG1, SNF5 and BAF170 to the *TCF* promoter and activates the Wnt signaling pathway.	Liver cancer	[46]
*NEAT1*	*NEAT1* interacts with BRG1 or BRM to form the paraspeckle structure.	Various human cancers	[41, 42]
*UCA1*	*UCA1* binds and represses the chromatin remodeling activity of BRG1, which promotes bladder cancer cell proliferation.	Bladder cancer	[35]
*HIF1A-AS1*	*HIF1A-AS1* interacts with BRG1, which contributes to regulate cell proliferation and apoptosis.	Lung cancer	[44, 45]
*Evf2*	*Evf2* represses the activity of BRG1 ATPase and chromatin remodeling.	Unknown	[43]

Furthermore, lncRNAs interact with the SWI/SNF complexes to affect tumorigenesis and development in different ways. For instance, a Wnt signaling pathway activating non-coding RNA *lncTCF7*, which recruits SWI/SNF to the promoter of TCF7, activates the Wnt signaling pathway by upregulating the expression of TCF7, which then promotes self-renewal of liver tumor stem cells [46]. LncRNAs can also recruit inflammatory transcription factor assembly into the SWI/SNF complexes. LincRNA *Cox2* promotes the transcription of late inflammatory genes in macrophages by regulating SWI/SNF mediated chromatin remodeling. Under the stimulation of bacterial LPS, lincRNA *Cox2* is required for the transcription of the late inflammatory response genes that regulate NF-κB. Specifically, lincRNA *Cox2* is assembled into the SWI/SNF complexes in cells after LPS stimulation, which causes the lincRNA *Cox2*-SWI/SNF complex to modulate the NF-κB subunits assembling into the SWI/SNF complexes. Therefore, SWI/SNF-related chromatin remodeling occurs in macrophages and causes the transcriptional regulation of the late response genes in the innate immune cells [48]. The immune system can regulate tumor progression and is one of the important mechanisms in tumorigenesis; because of this close relationship between the immune system and tumors, we hypothesize that there are many other kinds of lncRNAs that could assemble to SWI/SNF to recruit transcription factors, thereby affecting the progression of tumors and showing some form of inflammation regulation.

Some oncogenes also participate in the regulation of the SWI/SNF complexes in cancer. *MYC*, a multifunctional oncogene, plays a critical role in cell proliferation, differentiation, apoptosis, and genetic instability [49]. It can interact with different subunits of SWI/SNF. For example, the core subunit of SWI/SNF SNF5/INI1 interacts with *MYC* through the *MYC* basic helix loop helix (bHLH), leucine zipper (Zip) and the INI1 repeat 1 (Rpt1) domains and contributes to the transcription of MYC target genes [50]. BAF250A, a subunit of the SWI/SNF complexes, directly inhibits the expression of *MYC* in differentiating cells [51]. Methylated BAF155 (the core subunit of SWI/SNF complexes) can also be recruited to the *MYC* target gene *GADD45A* and promote cancer progression [49]. BRG1 is a tumor suppressor in most cancer types, but it promotes *MYC* transcription and maintains oncogenic programming in leukemia cells [52]. The transcriptional activation of *MYC* requires the interaction with the *MYC*-associated factor X gene (*MAX*), to form the *MYC-MAX* dimer [53]. The SWI/SNF complexes can also interact with *MAX*, for instance, BRG1 directly recruits to the *MAX* promoter and regulates the expression of *MAX* in lung cancer [54]. These studies provide evidence that an interaction between the SWI/SNF complexes and *MYC* is essential in cancer.

*KRAS*, first discovered in the rat sarcoma virus, plays a critical role in human cancer [55]. Knockdown of SWI/SNF subunits BRM, BRG1, hSNF5 and BAF250A decrease the activity of *KRAS* in colon cancer cells [56]. The Ras inhibitor RasGAP1 can also inhibit the BRM gene. BRG1 inactivating mutations may cooperate with *KRAS* mutations during carcinogenesis [57]. SNF5 can bind and activate the tumor repressor INK4A/ARF, which is in response to the oncogene *KRAS* [58]. Table 2 lists the various subunits of the SWI/SNF complexes associated with oncogenes such as *MYC* and *KRAS* in different cancer types. Understanding these relationships may provide insights into human genomic disorders, cell migration and cancer metastasis.

**Table 2:** The SWI/SNF complex affects cancer development by regulating oncogene expression

SWI/SNF	Interaction of SWI/SNF with oncogene	Cancer types/Phenotypes	Refs
BRG1	BRG1 promotes myc transcription and maintenance of oncogenic programming.	Leukemia	[52]
BRG1	BRG1 directly recruits to the *MAX* (myc-associated factor X gene) promoter and regulates the expression of *MAX*.	Lung cancer	[54]
SNF5/INI1	SNF5/INI1 interacts with c-myc and recruits the SWI/SNF complex, which contributes to the transcription of *MYC* target genes.	Apoptosis	[50]
BAF250A	BAF250A subunit directly inhibits the expression of *MYC*.	Differentiation associated cell cycle arrest	[51]
BAF155	Methylated BAF155 recruited to *MYC* target gene, *GADD45A*.	Breast cancer progression and metastasis	[49]
BRG1	BRG1 inactivating mutation may cooperate with *KRAS* mutation during carcinogenesis.	Lung Cancer	[57]
BRM, BRG1, hSNF5, BAF250A	The RAS inhibitor RasGAP1 can inhibit the BRM, knockdown SWI/SNF members BRM, BRG1, hSNF5 and BAF250A, and decrease the active *KRAS*	Colon cancer cell	[56]
BRG1	BRG1 inactivating mutation cooperates with oncogenic *KRAS* and promote the progression of pancreatic ductal adenocarcinoma.	Pancreatic ductal adenocarcinoma	[59]
SNF5	SNF5 can bind and activate the tumor repressor INK4A/ARF, which is in response to oncogene *KRAS*, indicating that the function of SWI/SNF link to oncogene *KRAS*.	Lung tumor	[58]

## Conclusion

**Fig. 3:**
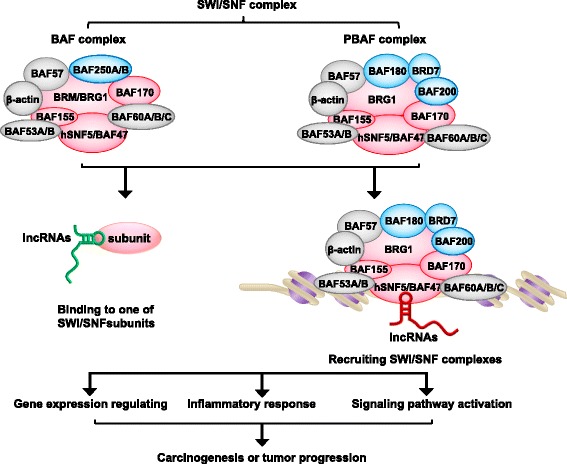
Model of lncRNAs interact with SWI/SNF. Two subclasses of the SWI/SNF complexes, BAF and PBAF have a diverse composition of the subunits. These subunits interact with lncRNAs, which bind to or recruit chromatin and make distinctive contributions to regulate chromatin remodeling and oncogene expression

In this review, we have summarized different subunits of the SWI/SNF complexes. These subunits recruit to chromatin for chromatin remodeling and transcriptional control through interactions with lncRNAs (Fig. 3). This review gave a detailed description of the function of the SWI/SNF complexes and the relationship between the SWI/SNF complexes and lncRNAs, which will offer new insights into the processes of cancer and provide novel therapeutic approaches to SWI/SNF-mutant cancers.

## Abbreviations

ARPs: Actin-related protein
ATP: Adenosine triphosphate
BAF: BRG/hBRM associated factors
bHLH: Basic helix loop helix
BRG1: BRM related gene 1
BRM: Brahma
Bromo: Bromodomain
CHD: Chromodomain helicase DNA binding
DSB: DNA double strand break
HO: Homothallic switching endonuclease
INO80: Inositol requiring 80
ISWI: Imitation switch
LncRNAs: Long noncoding RNAs
MAX: Myc-associated factor X gene
NEAT1: Nuclear paraspeckle assembly transcript 1
NSCLC: Non-small lung cancers
PBAF: Polybromo associated BAF
Rpt1: INI1 repeat 1
SChLAP1: Second chromosome locus associated with prostate-1
SUC2: Sucrose invertase
SWI/SNF: Switching defective/sucrose nonfermenting
Zip: Leucine zipper
